# Mouth-Opener Technique for Ultrasound-Guided Inferior Alveolar Nerve Block: A Case Series of Extraction of Impacted Mandibular Third Molars

**DOI:** 10.7759/cureus.44179

**Published:** 2023-08-26

**Authors:** Daisuke Oiwa, Sho Kumita, Tomohiro Chaki, Satoshi Ono

**Affiliations:** 1 Department of Dental Anesthesiology and Perioperative Management, Hinode Makomanai Dental Hospital, Sapporo, JPN; 2 Department of Anesthesiology, School of Medicine, Sapporo Medical University, Sapporo, JPN

**Keywords:** pain management, ultrasound guidance, impacted mandibular third molars, mouth opener, inferior alveolar nerve block

## Abstract

Extraction of the impacted mandibular third molar (IMTM) is common in oral surgery, but its postoperative pain is severe. Ultrasound-guided inferior alveolar nerve block (UGIANB) is an analgesic technique in the mandibular nerve region. We describe UGIANB using a mouth opener and report the cases with a good postoperative course.

Six patients underwent the extraction of bilateral IMTMs under general anesthesia. After surgery, we performed UGIANB and administered 5 mL of 0.375% levobupivacaine on each side. The postoperative numerical rating scale pain scores were 1 (0-2) and 2.5 (0-5) (mean (range)), postoperative day one and seven, respectively. The postoperative quality of recovery-40 scores were 188.5 (8.1) and 191.7 (7.6) (mean (SD)), postoperative day one and seven, respectively. No procedural complications were encountered.

We performed UGIANB with a mouth opener on a patient with IMTM extraction and were able to provide safe and good analgesia.

## Introduction

Extraction of the impacted mandibular third molar (IMTM) is the most frequent oral and maxillofacial surgical procedure. The worldwide prevalence of IMTMs is 25% [1]. The major complication of its extraction is postoperative pain, which can persist for more than a week despite analgesic therapy, and this pain induces negative impact of the patient’s health and quality of daily life [2-7]. For this pain management, the intraoral inferior alveolar nerve block (IANB) is one of the most common techniques in dentistry [6-10]. With this traditional block, the needle tip is blindly placed toward the mandibular sulcus or lingula, with a success rate of 75-85% and a blood aspiration incidence rate of 2.9-22% [6,7]. Ultrasound-guided inferior alveolar nerve block (UGIANB) approaches the same area as the conventional IANB from the body surface. In recent years, it has shown high success rates and effectiveness in mandible surgery [11,12]. UGIANB is useful because it allows real-time monitoring of the needle tip, the important structures such as the arteries and the muscles, and the local anesthetic. In this technique, the probe remains below the zygomatic arch, and the patient's mouth is requested to stay wide open for pulling the mandible downward and delineating the target space. However, we often encounter clinical situations in which the target space is difficult to visualize in small aperture patients. Currently, it is difficult to perform the UGIANB in patients with unstable apertures. We described UGIANB using a mouth opener, which enabled us to steadily delineate a wide target area and report the cases in which the postoperative pain of IMTM extraction was effectively controlled.

## Case presentation

This case report was approved by our institutional research ethics committee (approved code: 22-2), and written case report consent was obtained from all patients. Six patients underwent the bilateral IMTM extraction under general anesthesia between April and September 2022 at Hinode Makomanai Dental Hospital. Every patient was in good health, with the American Society of Anesthesiologists physical status classification level I, and did not receive any medication. Every patient had no previous experience of adverse reactions to any of the local anesthetics utilized in this report. There were no significant problems with the patients' airway assessment; all had the Mallampati class I, could open their mouths more than three lateral fingers, and had good cervical mobility. After discussing with the surgeon, the expected possibilities of aspiration, operative time, water infusion, bleeding and postoperative pharyngeal pain, and airway management devices were decided individually. Each patient underwent preoperative panoramic radiography and showed bilateral IMTMs with at least one impaction categorized as class II-B in accordance with the Pell and Gregory categorization [13]. Four of the six patients additionally extracted maxillary premolars and third molars along with the IMTMs. The anatomical characteristics of IMTMs and patients' background characteristics and perioperative data are summarized in Table 1.

**Table 1:** Demographic and perioperative data. F, female; M, male; SGA, supraglottic airway device; LP, loxoprofen sodium hydrate 60 mg; AA, acetaminophen 1000 mg. Patients 1, 2, 3, and 5 underwent additional removal of one or two maxillary third molars, and patient 5 underwent removal of four premolars.

	Age/Sex	Height(cm)/Weight(kg)/BMI (kg m^-2^)	Airway management	Pell and Gregory categorizations		Operating time/ Anesthesia time (min)	Number of teeth removed	Local anesthetic (mL)	Oral medication after surgery
				Right	Left				
1	18/F	153/60/26	SGA	IIB	IA	31/52	3	6.6	LP
2	26/F	158/53/21	intubation	IIB	IA	46/78	4	6.8	AA
3	31/F	155/50/21	SGA	IIB	IA	30/39	4	9	AA
4	27/M	174/71/23	SGA	IB	IIB	14/32	2	5.3	AA
5	20/F	164/56/21	intubation	IIB	IB	58/80	8	12.6	AA
6	41/F	167/68/24	intubation	IIB	IIB	36/57	2	3.6	LP

Propofol and remifentanil were used to induce general anesthesia. Using a target-controlled infusion pump and a bispectral index monitor, propofol was infused at 3-4 mcg mL-1 and remifentanil was infused at a rate of 0.2 mcg kg^-1^ min^-1^ for the induction. Three of the six patients underwent orotracheal intubation (spiral tube; Fuji System Japan, Tokyo, Japan) with the administration of rocuronium at 0.6 mg kg^-1^. The other three of the six patients underwent supraglottic airway insertion (AuraOnceTM; Ambu, Copenhagen, Denmark) without using a neuromuscular blocking agent.

Local infiltration anesthesia was a 2% lidocaine with 1:80,000 adrenaline preparation to prevent bleeding, and 1.8 mL was administered per tooth extraction. All tooth extractions were performed by the conventional method. The mucoperiosteal flap extended from the mesial corner of the second molar distally to the retromolar region. Bone was removed using both a round and a fissure bur in a high-speed handpiece. The tooth was sectioned by the fissure bur and dislocated and extracted using an elevator. The wound was irrigated with a physiologic saline solution. A 4-0 nylon suture was used to close the wound without tension.

**Figure 1:**
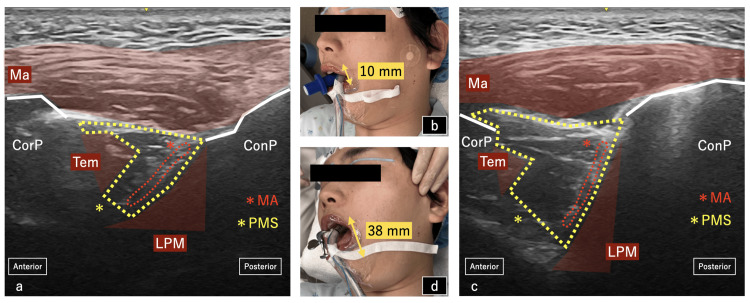
Changes in appearance and ultrasound imaging with the use of a mouth opener. a) The ultrasound image without a mouth-opener position. The yellow dotted line shows the pterygomandibular space and the red dotted line shows the maxillary artery. The pterygomandibular space is narrow and has the risk of puncturing the maxillary artery. b) Insertion of the bite-blocker with 10mm between upper and lower incisors. c) The ultrasound image with the mouth-opener position. The pterygomandibular space is more expansive than without the mouse-opener position and reduces the risk of puncturing the maxillary artery. d) Insertion of the mouth-opener with 38 mm between upper and lower incisors. PMS, pterygomandibular space; MA, maxillary artery; Ma, masseter; Tem, temporalis; LPM, lateral pterygoid muscle; ConP, condylar process; CorP, coronoid process.

After all surgical procedures, UGIANB was performed under general anesthesia in all cases (DO). Local infiltration anesthesia was performed preoperatively. To reduce the risk of local anesthetic systemic toxicity (LAST), UGIANB was performed under general anesthesia postoperatively. A mouse opener was inserted and opened as much as possible, maintaining an interincisal distance of at least 30 mm. The high-frequency linear probe (Venue go; GE healthcare, Chicago, USA) was positioned transversely below the zygomatic arch. A mouth opener (Almighty Mouth Gag; YDM Japan, Tokyo, Japan) was inserted (Figure 1).

This force shifted the condylar process forward and downward, widening the pterygomandibular space (PMS) through which the mandibular nerve branches pass. A 22-G, 5-cm echogenic needle (SonolectNeedle USG-type corner cube reflector; Hakko Co., Nagano, Japan) was inserted from the cranial side of the probe using the out-of-plane technique. Ultrasound images delineated the PMS as a cleft surrounded by the mandible and the masseter laterally, and the pterygoid muscles medially from anatomical standpoint [7]. We administered a small amount of local anesthetic once the needle tip was seen to pass beyond the masseter muscle. If the entire lateral pterygoid muscle was seen to have been pushed down by local anesthetic, we took this as a sign of a successful procedure. We administered 5 mL of 0.375% levobupivacaine on each side. After the block was completed, the patient awoke from anesthesia and exited the operating room.

**Figure 2:**
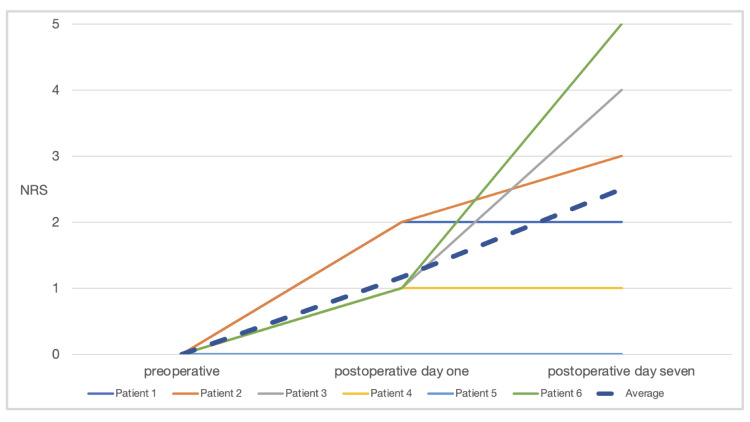
Perioperative numerical rating scale (NRS) pain scores.

Two of the six patients received a loxoprofen sodium hydrate 60 mg every eight hours for three days and the other four of the six patients received acetaminophen 1000 mg every eight hours for three days after dinner on the day of surgery. Loxoprofen sodium hydrate 60 mg was administered as a rescue analgesic in response to the patient’s request. All patients received a cefditoren pivoxil tablet for antibiotics 100 mg every eight hours for three days from after dinner on the day of surgery. Pain intensity was assessed using the numerical rating scale (NRS; 0-10), and the quality of postoperative recovery was recorded using the quality of recovery (QoR)-40 score (40-200) [14]. NRS and QoR-40 assessments were conducted preoperatively, postoperatively day (POD) one, and POD seven, with POD one marking the time of discharge 24 hours after surgery, and POD seven marking the outpatient visit for suture removal. All patients reported slight pain (NRS of POD one was 2/10 or less). Only one patient required 60 mg of loxoprofen for rescue analgesic at 210 minutes after surgery. No procedural complications were encountered. Although three of the six patients reported post-procedural discomfort on POD one, all responded to minor analgesics. Two of the six patients reported sore throats on the day of surgery, but this symptom disappeared by POD one (Figure 2; Tables 1, 2).

**Table 2:** Perioperative QoR-40 scores. GS, global score; PC, physical comfort; PI, physical independence; ES, emotional state, PS, psychological support; QoR, quality of recovery.

	Patient 1	Patient 2	Patient 3	Patient 4	Patient 5	Patient 6	Mean±SD
Preoperative							
GS (40–200)	195	195	187	200	194	192	193.8±4.3
PC (12–60)	58	60	58	60	59	60	59.2±1.0
PI (5–25)	25	24	25	25	25	25	24.8±0.4
ES (9–45)	42	41	36	45	42	44	41.7±3.1
PS (7–35)	35	35	33	35	33	28	33.2±2.7
Pain (7–35)	35	35	35	35	35	35	35.0±0.0
Postoperative day one							
GS	194	177	192	197	191	180	188.5±8.1
PC	57	49	57	60	57	57	56.2±3.7
PI	24	21	25	24	21	19	22.3±2.3
ES	45	44	45	45	45	42	44.3±1.2
PS	35	35	35	35	34	30	34.0±2.0
Pain	33	28	30	33	34	32	31.7±2.3
Postoperative day seven							
GS	199	186	191	199	195	180	191.7±7.6
PC	60	58	57	60	57	54	57.7±2.3
PI	25	22	25	25	25	22	24.0±1.5
ES	45	42	42	45	45	41	43.3±1.9
PS	35	35	35	35	35	32	34.5±1.2
Pain	34	29	32	34	33	31	32.2±1.9

No severe complications, such as blood aspiration, were encountered.

## Discussion

**Figure 3:**
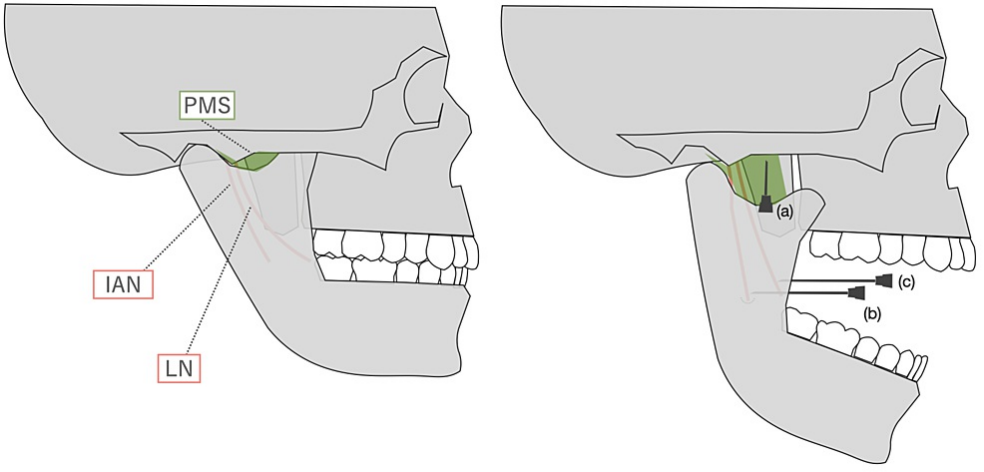
Anatomical structures in closed (left) and opened (right) mouth positions. a) The mouth-opener technique for ultrasound-guided inferior alveolar nerve block (UGIANB). The mouth-opener device makes it easier to maintain the maximum opening position and allows for a larger pterygomandibular space (PMS) to be visualized with ultrasound. b) The conventional intraoral inferior alveolar nerve block (IANB) technique. The needle is blindly inserted above the mandibular sulcus or lingula. c) The anterior intraoral IANB technique. The needle is blindly inserted more anteriorly than in the conventional intraoral IANB technique. The lingual nerve injury is at risk with this technique.

Intraoral IANB is the conventional nerve block used in dentistry [6-10]. Various approaches have been reported in recent years, but the possibility of failure and blood aspiration, with a success rate of 75-85% and incidence rate of 2.9-22%, has been pointed out due to the blind technique (Figure 3).

In contrast, the UGIANB is superior in allowing real-time monitoring of the positional relationship between the needle and the various tissues. We demonstrated that UGIANB could administer anesthetics into the PMS accurately in the manual opening position by cadaver [15]. However, we often encounter clinical situations in which the target space is difficult to visualize in small aperture patients. In the limited PMS visualization, the maxillary artery may be in close area to the position of the needle insertion (Figure 1a). Otherwise, the mandibular condylar process moves forward and downward when the mouth is opened. It makes the PMS visualization broader by using ultrasound. In the maximum opening position with the mouth opener, the angle of the PMS created by the masseter muscle and lateral pterygoid muscle increases compared to when the mouth is closed, and the target PMS can be widely visualized by ultrasound (Figure 1c). Compared to manual opening, the maximum opening position is maintained stably. Our techniques are likely to reduce the difficulty of the procedure and the incidence of complications such as maxillary artery puncture. However, the use of ultrasound requires more expertise compared to blind one.

Many studies have examined postoperative analgesic management for this surgery, using methods such as fixed-dose ibuprofen and acetaminophen [2], local application of bupivacaine [3], and light irradiation with a low-level laser or light-emitting diode [4]. However, no gold standard has yet been established for this surgery. An investigation of postoperative pain following the extraction of IMTMs in a Japanese population revealed that the highest pain intensity (visual analog scale, 0-100) on POD one was 84.8 ± 15.8 (mean ± SD) under local anesthesia [9]. According to these findings, Japanese patients who underwent this procedure experienced moderate to severe postoperative pain. Our patients' median NRS on POD one was 1 (range, 0-2).

The QoR-40 score is a recovery-specific, patient-rated questionnaire that contains 40 items in five sub-scales: physical comfort; physical independence; emotional state; psychological support; and pain [14]. Myles et al. recently reported that the minimal clinically significant difference, as a score change of 6.3 in the global QoR-40 score, could indicate a meaningful change in health status [16]. Our six cases showed less degradation of the global QoR-40 score from baseline to POD one, with a mean difference of 5.3. This suggests that patients experienced no clinically important change in status. The novel UGIANB may provide excellent quality postoperative recovery. QoR-40 scores for POD seven were the same or higher than on POD one in five of the six patients. This indicates that the patient’s quality of life had recovered toward the preoperative baseline. These findings suggest that UGIANB may contribute to acute postoperative analgesia and recovery in IMTM extraction patients. Furthermore, the mouth-opener technique presented here may improve the certainty and safety of UGIANB.

We performed UGIANB after surgical extraction in consideration of the risk of LAST. Also, anatomical deviations and tissue swelling generally make performing nerve blocks difficult postoperatively. However, the tooth extraction procedure is short and does not cause significant anatomical changes, the difficulty of the nerve block remains the same as preoperatively.

The maxillary nerve block was not performed for maxillary third molar extractions to reduce the total dose of local anesthetic. Several studies have included maxillary third molar extraction when investigating postoperative pain after IMTM extraction [2,5]. However, maxillofacial pain might arrive in the early postoperative period because UGIANB targets only the inferior alveolar, lingual, and buccal nerves. In the future, we are considering performing the maxillary nerve block simultaneously as UGIANB.

## Conclusions

We report several IMTM extraction cases in which a mouth opener was useful in UGIANB. Using the mouth opener allows clear delineation of the pterygomandibular space under ultrasound guidance, which may contribute to the safety and certainty of nerve block. However, the use of ultrasound requires more expertise compared to blind conventional block.

We performed UGIANB after surgery considering the risk of LAST and did not perform the maxillary nerve block for the extraction of maxillary third molars. The investigation of the total quantity of anesthetics and timing of UGIANB using the maxillary nerve block was done at the same time.
